# Supplementary material for: Identification and Functional Analysis of the fruitless Gene in a Hemimetabolous Insect, Nilaparvata lugens
Source: Insects. 2024 Apr 11;15(4):262. doi: 10.3390/insects15040262 (PMC11050625; doi:10.3390/insects15040262)
Supplement: Supplementary file 1 [file insects-15-00262-s001.zip › insects-2873428-supplementary.pdf]

Supplementary Materials:

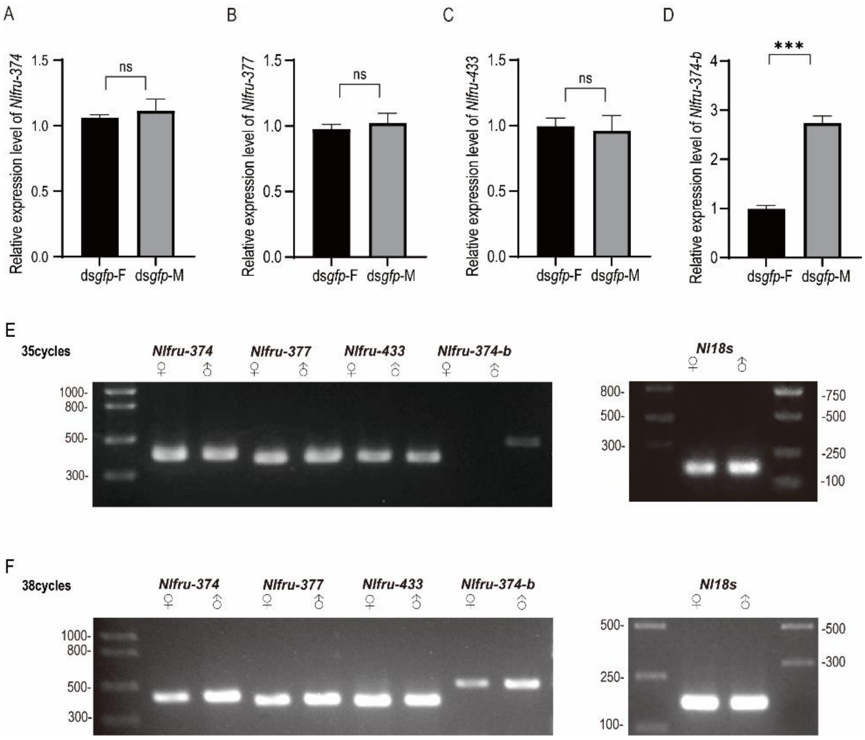

Figure. S1. The expression of different alternative spliceosomes in male and female insects. A, B, C, D) Expression levels of Nlfru-374, Nlfru-377, Nlfru-433, Nlfru-374-b in females and males. We did 3 replications of each experiment, each containing 10 BPHs. E, F) Sex-biased expression of four NlFRU splice variants. In RT-PCR, sex-specific cDNA was used as a template and specific primers were used for 35 and 38 cycles, respectively, to identify their respective 4 splice variants. Primer Nl*18s* RNA as a positive control.

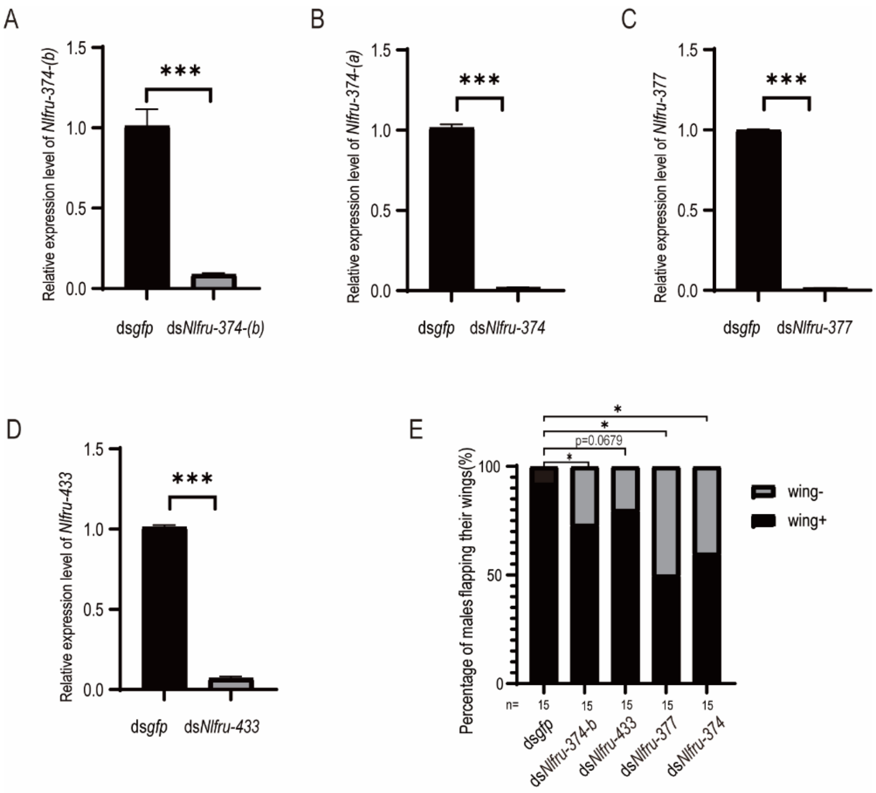

Figure. S2. Silencing efficiency of specific RNAi for different alternative spliceosomes and the proportion of males flapping their wings during courtship. A)-D）Expression of *Nlfru* after RNAi-specific selective spliceosome. We did 3 replications of each experiment, each containing 10 BPH. E) Proportion of males flapping their wings during courtship after RNAi on specific alternative spliceosomes, ds*Nlfru-374-b*, ds*Nlfru-377*, ds*Nlfru-374* had an obvious influence on the male's wing flapping during the mating behavior, and the p-value between ds*Nlfru-433*-treated males and ds*gfp* males was 0.0679. n represents the number of groups. (Student's t-test is used; **, P < 0.01; Chi-square test is used, *, P < 0.01)

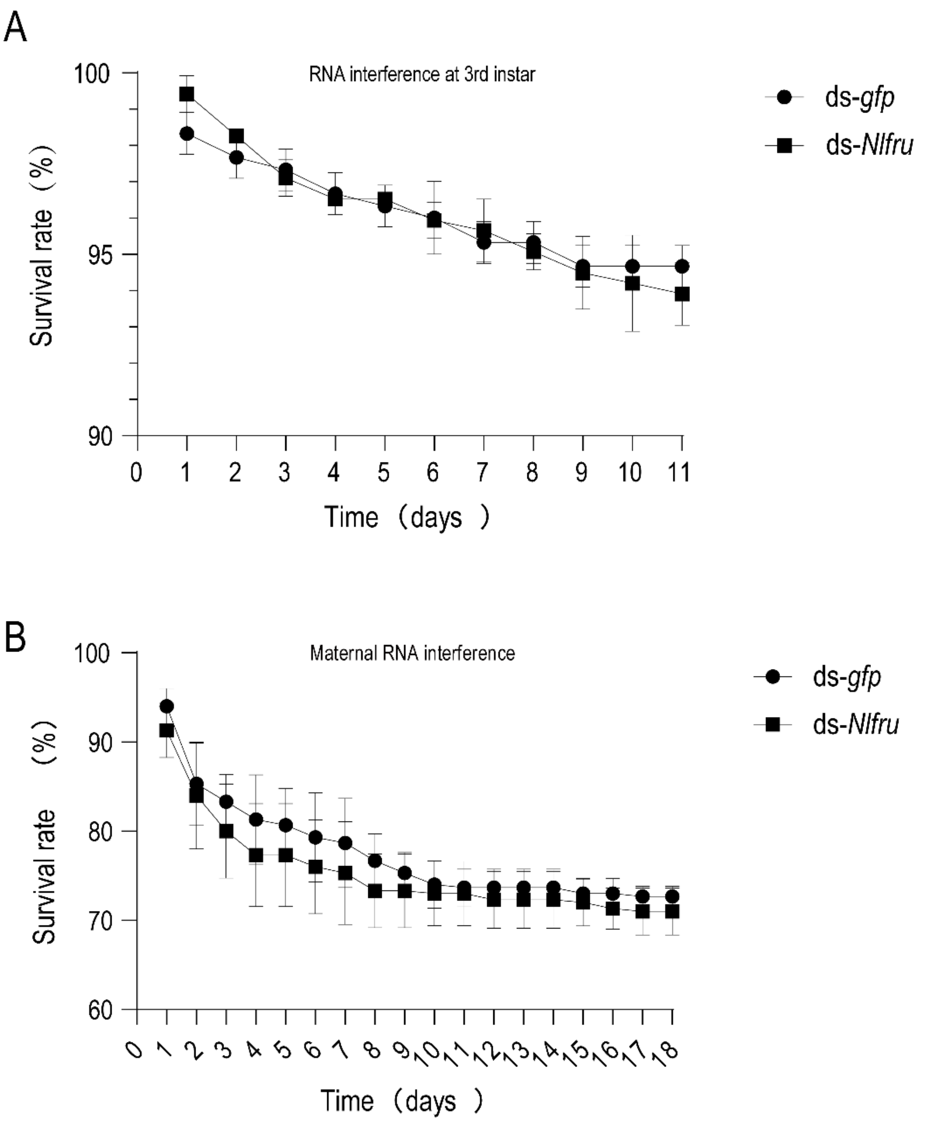

Figure S3 The survival rate of BPHs, which injected with dsNlfru in 3rd instar (A) and newly emerged females (0-1st hour) (B). Perform three replications for each group. n represents the number of BPH in each repetition. 3 replications were used in each experiment, and every replication contained 90 BPHs.

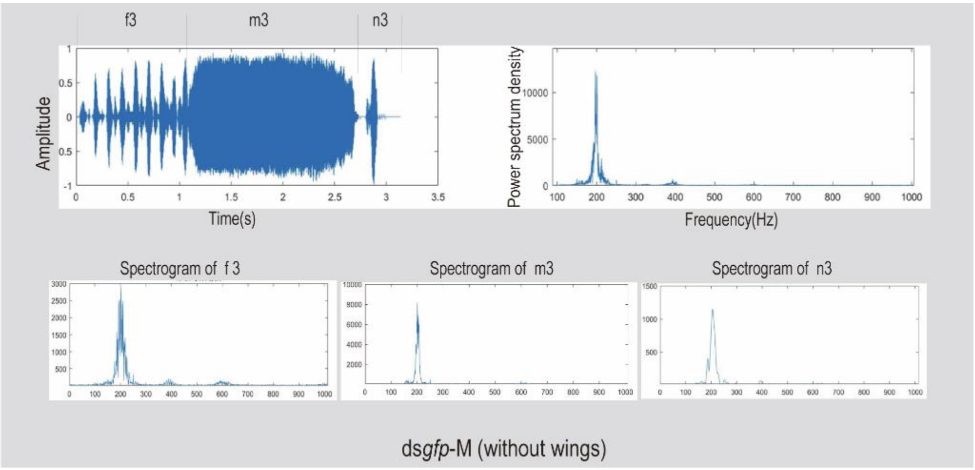

Figure S4. Spectra and spectra of courtship signals in wild-type wingless males.

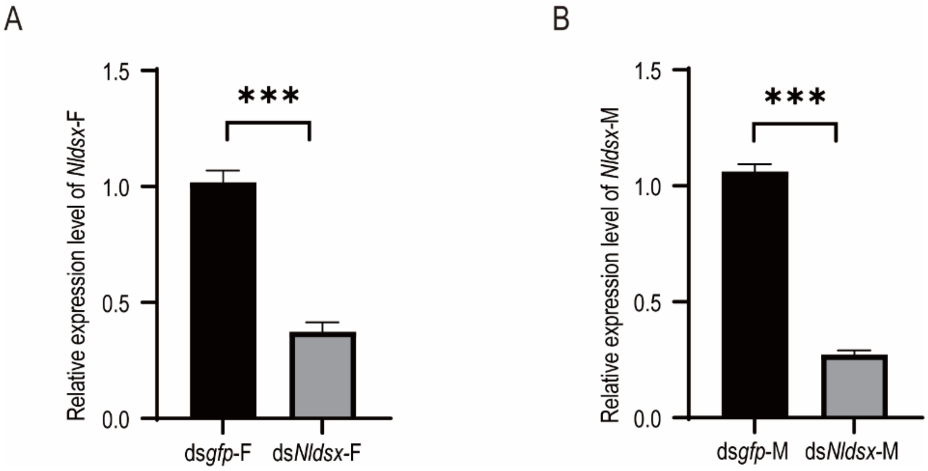

Figure. S5. Interference efficiency of ds*Nldsx*. A) Expression of *Nldsx* in female BPH after knockout of the *Nldsx* common segment. B）Expression of *Nldsx* in male BPH after knockout of the *Nldsx* common segment.We did 3 replications of each experiment, each containing 10 BPH.

Table S1. The main frequency of the courtship song.

Sample	#1 (Hz)	#2 (Hz)	#3 (Hz)	#4 (Hz)	#5 (Hz)	p-value
WT-F	250	240	200	210	220	
ds*Nlfru*-F	200	250	240	250	260	0.286144559
WT-M	200	200	230	220	240	
ds*Nlfru*-M	220	200	210	220	270	0.689723267

The main frequency of the courtship in each group was compared: the p-value of WT-F and dsNlfru-F was 0.286144559, and the p-value of WT-M and dsNlfru-M was 0.689723267. (Student's t-test is used.)

Table S2. The sequences of primers.

Primer name	Sequence
Nlfru-374-b-F（499）	GGTGCTAAACTAGGTTGCCG
Nlfru-374-b-R（499）	CTGGGCAGAGAATGGTGGTC
Nlfru-374-F（426）	*TGCCCTAAAGTGCCTAAAACG*
Nlfru-374-R（426）	*GAGCTTAACTAGACACCGACA*

Nlfru-377-F（395）	ATTTGGAAAATGACCAGATT
Nlfru-377-R（395）	ATGTCCAGCCAAAAATGCGG
Nlfru-433-F（387）	TTGCTCGTTTATCGCTTGCC
Nlfru-433-R（387）	TTTCGTGGCTTGACTTGTGC
ds*Nlfru*-c-F	T7+AGGACTAGCTGACTCGAGGAC
ds*Nlfru*-c-R	T7+ACTGTTTGCCCTTCGGACTC
ds*Nlfru*-433-F	T7+GCTCTCAGATCCAGCGCTAC
ds*Nlfru*-433-R	T7+TCAGGCACGAAACTGCCATA
ds*Nlfru*-377-F	T7+TCCAGCCAAACCCAAAGAGAT
ds*Nlfru*-377-R	T7+ATTGCCACAGTAGGGGCATTT
ds*Nlfru*-374-F	T7+GCACATGCGTCTGACATTCG
ds*Nlfru*-374-R	T7+TTGCTTATCGGCTGAGGAGG
ds*Nlfru*-374-b-F	T7+ACCTTTTCAACATGGCTTGATT
ds*Nlfru*-374-b-R	T7+CGGCAACCTAGTTTAGCACC
ds*gfp*-F	T7+CGCGCCGAGGTGAAGTTC
ds*gfpR*	T7+GTTCACCTTGATGCCGTTC
Q-Nl18S-F	GTAACCCGCTGAACCTCCT
Q-Nl18S-R	TCCGAAGACCTCACTAAATC
Q-*Nlfru*-c-F	ACGGAGAAGTCAATGTCAAGCA
Q-*Nlfru*-c-R	TTTGTTCGACTGAGGAGCGG
Q-*Nlfru*-433-F	CAGATGTCGAGTCCGAAGGG
Q-*Nlfru*-433-R	CGTAGCGCTGGATCTGAGAG
Q-*Nlfru*-377-F	TGGAAAATGACCAGATTTGCGAC
Q-*Nlfru*-377-R	CTGCTACGTACGGACTTGGA
Q-*Nlfru*-374-F	ATGACCAGATTTGCGACATGC
Q-*Nlfru*-374-R	TCGGACACACAAATCTGCCT
Q-*Nlfru*-374-b-F	TCATCATAGCAAGGGAAA
Q-*Nlfru*-374-b-R	CTCAGCACATCAGTCAAA
Q-*Nl-flightin*-F	TGGATAAACGCAACAAAGG
Q-*Nl-flightin*-R	GATGTAGTCAATCGGCATG
ORF-*Nlfru*-374-b-F	TGCTAAACTAGGTTGCCGGAT
ORF-*Nlfru*-374-b-R	CCACCAGCGGGTTGATCAGTG
ORF-*Nlfru*-374-a-F	CCGTTGTCCTCTCCTCTCTAA
ORF-*Nlfru*-374-a-R	CCAGCGGGTTGATCAGTGAAT
ORF-*Nlfru*-377-F	AAGGGACCGTTGTCCTCTCC
ORF-*Nlfru*-377-R	GGACAGAAAGGCGGTATTGC
ORF-*Nlfru*-433-F	ACCGTTGTCCTCTCCTCTCTAA
ORF-*Nlfru*-433-R	TGCTAGGTAGGAGGCAGGTAG
